# Shape-based peak identification for ChIP-Seq

**DOI:** 10.1186/1471-2105-12-15

**Published:** 2011-01-12

**Authors:** Valerie Hower, Steven N Evans, Lior Pachter

**Affiliations:** 1 Department of Mathematics, University of California, Berkeley, California, USA; 2 Department of Statistics, University of California, Berkeley, California, USA; 3 Department of Molecular and Cell Biology, University of California, Berkeley, California, USA

## Abstract

**Background:**

The identification of binding targets for proteins using ChIP-Seq has gained popularity as an alternative to ChIP-chip. Sequencing can, in principle, eliminate artifacts associated with microarrays, and cheap sequencing offers the ability to sequence deeply and obtain a comprehensive survey of binding. A number of algorithms have been developed to call "peaks" representing bound regions from mapped reads. Most current algorithms incorporate multiple heuristics, and despite much work it remains difficult to accurately determine individual peaks corresponding to distinct binding events.

**Results:**

Our method for identifying statistically significant peaks from read coverage is inspired by the notion of persistence in topological data analysis and provides a non-parametric approach that is statistically sound and robust to noise in experiments. Specifically, our method reduces the peak calling problem to the study of tree-based statistics derived from the data. We validate our approach using previously published data and show that it can discover previously missed regions.

**Conclusions:**

The difficulty in accurately calling peaks for ChIP-Seq data is partly due to the difficulty in defining peaks, and we demonstrate a novel method that improves on the accuracy of previous methods in resolving peaks. Our introduction of a robust statistical test based on ideas from topological data analysis is also novel. Our methods are implemented in a program called T-PIC (Tree shape Peak Identification for ChIP-Seq) is available at http://bio.math.berkeley.edu/tpic/.

## Background

With rapidly decreasing costs of sequencing, next-generation sequencing assays are increasingly being used for molecular measurements [1]. These techniques generate millions of short reads and massive data sets, making it computationally challenging to properly analyze the data. One such assay, called ChIP-Seq (chromatin immunoprecipitation followed by sequencing), is used to determine DNA binding sites of a protein (see [2,3] for a review). In ChIP-Seq, protein is first cross-linked to DNA and the fragments subsequently sheared. Following a size selection step that enriches for fragments of specified lengths, the fragments ends are sequenced, and the resulting reads are aligned to the genome. Reads pile up at bound regions referred to as "peaks", but due to mapping challenges and biases in various aspects of existing protocols, identifying peaks is not a straightforward task.

While there are many current algorithms for analyzing ChIP-Seq data (see [4] for a recent review), there is still room for improvement as most rely on adhoc heuristics including coverage thresholds and poorly motivated filters. In particular, while existing methods rely on depth of coverage to determine likely binding sites using statistical methods, the determination of *regions *of binding, i.e. peak boundaries, is frequently based on heuristics.

We present a novel approach for calling peaks that is based on evaluating the significance of a robust test statistic that measures the extent of pile-up of reads. Specifically, we use define and evaluate the "shape" of putative peaks to differentiate between random and nonrandom fragment placement on the genome. We compare our predictions to two state-of-the-art methods (based on comparisons in [4,5]) using two published data sets and demonstrate improved performance.

## Results and Discussion

### Algorithm

#### Overview of the algorithm

The input to our algorithm consists of the aligned reads for both the sample and input control. We create a 'coverage function'--a map *f *from the genomic coordinates to the non-negative integers-- by extending each of the aligned sample reads to the average fragment length *L*. The 'height' *f(t) *at a nucleotide *t *is the number of such extended reads that contain *t*. This piecewise constant function is the data that we analyze.

We will flag peaks in the coverage function that are, in a suitable sense, 'anomalous' as being likely protein binding sites. In order to turn this some-what vague idea into a well-founded statistical inference procedure we require two basic ingredients. Firstly, we need a numerical test statistic that measures some feature of a peak such that peaks which result in extreme values of the test statistic might reasonably be expected to be binding sites. Secondly, in order to calibrate whether a value of the test statistic is so large that it is difficult to explain as simply being the consequence of random fluctuations (and thus indicates the presence of a binding site) we need a stochastic model of the coverage function for the 'null' situation when we are in a region of the genome that doesn't contain a binding site.

#### A tree shape statistic ℳ to measure "peakness"

The most obvious test statistic is simply the height of a peak. However, such a statistic reflects the depth of coverage at a single site, and ignores valuable information in the form of the coverage depth in the neighboring region. Motivated by current work in topological data analysis (TDA) [6], we propose the following more synoptic measure of a peak's *shape *that incorporates information in the neighborhood of each site and therefore allows for defining binding regions, and not just sites.

Suppose we have an interval [*a*, *b*] of the genome that corresponds to an *excursion *of the coverage function above some height *h*. That is,

$$\begin{array}{l} f(a)=f(b)=h,\;\text{and}\\ f(t)>h\;\text{for}\;a\text{<}t\text{ <}b \end{array}$$

**Figure 1:**
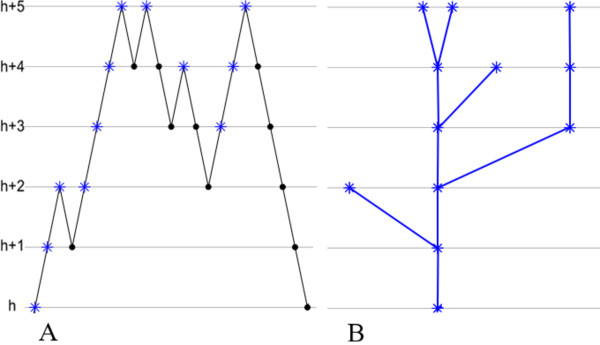
**A lattice path excursion and its associated tree**. An example of lattice path excursion (A) and its associated rooted tree (B) is given. The rooted tree is obtained by taking equivalence classes of vertices in (A), as explained in [7,8]. The vertices in (A) that are chosen representatives for the equivalence classes are depicted with blue stars.

Let *a *= *t*_0 _<*t*_1 _< ... <*t_n _*= *b *be the locations at which the coverage function changes value. It will typically be the case that the jump *f(t_k_) *- *f*(*t*_*k *- 1_) at the location *t_k _*is either +1 (when *t_k _*is the start of a single read) or -1 (when *t_k _*is the end of a single read under our Specification that all reads are taken to have length *L*). The sequence of integers {*f*(*t*_0_), *f*(*t*_1_), ..., *f*(*t_n_*)} is then a *lattice path *that begins and ends at the level *h *and exceeds *h *else-where. As illustrated in Figure 1 and discussed more formally in [7,8], there is bijection between lattice path excursions (starting and ending at height *h*) and rooted trees with root at height *h*. The tree captures in compact form the important features of the excursion of the coverage function.

**Figure 2:**
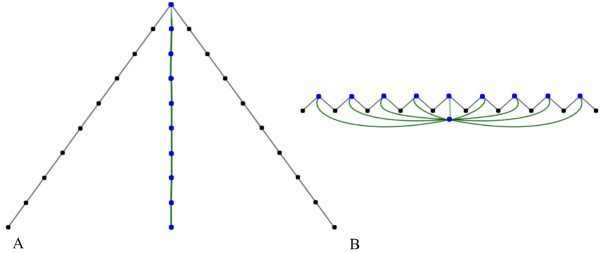
**Two extremal trees**. Two extremal trees are represented--the path *P*_10 _(A) and star graph *S*_10 _(B) on 10 vertices (blue vertices and green edges)--together with the jump skeleta (black vertices and edges) that give rise to the trees.

We need to further summarize this tree using an appropriate numerical statistic. In order to motivate our choice, consider the extreme cases of the trees that could arise. Figure 2 depicts the lattice paths and corresponding rooted trees for, respectively, a perfect peak and perfect noise. For a tree with *n *vertices, we look for a statistic that attains its greatest and least values, respectively, on the path *P_n _*and the star *S_n_*. A *matching *of a tree *T *is a subset *M *of the edges of *T *with the property that no two edges in *M *share a common vertex of *T*. A matching *M *is *maximal *if it contains at least as many edges as any other matching. We define ℳ(*T*) to be the number of edges in a maximal matching for the tree *T*. Note that $ℳ\left(T\right)\leq \left\lfloor \frac{n}{2}\right\rfloor =ℳ\left(P_{n}\right)$ and ℳ(*T*) ≥ 1 = ℳ(*S_n_*) for any tree *T *with *n *vertices. In general, excursions of the coverage function that correspond to sharp peaks result in tall, spindly trees with large values of ℳ(*T*), whereas broad, spreadout peaks result in low, bushy trees with small values of ℳ(*T*). In our implementation, we calculate the tree shape statistic ℳ using the algorithm in [9].

#### A null stochastic model of the coverage function

Following [7], we model the 'null' or 'background' placement of read starting locations in some region of the genome as a homogeneous Poisson process with rate *ρ*. That is, we replace the discrete set of nucleotide positions by a continuous interval and suppose that the distances between the starts of successive reads are independent random variables that each have an exponential distribution with mean $\frac{1}{\rho }$. The value of the coverage function at some position *t *is then just the number of points of the Poisson process that happen to fall in the interval [*t *- *L*, *t*]. This random variable has a Poisson distribution with mean *θ *= *ρL*; that is, the mean height of the coverage function at any fixed location is *θ*.

Even in the absence of binding, some genomic regions systematically receive a large number of fragments while others receive very few [10]. Hence, it would be inappropriate to use the same rate *ρ *for the entire genome and it is necessary to first divide the genome into regions across each of which we expect the background to be homogeneous and assign an individual rate to each one. We describe our procedure for determining these regions and estimating the local rates later.

The following consequences of this Poisson null model are established in [7].

Firstly, the random lattice path produced by recording the successive values of the coverage function at locations where it changes values (that is, where a read begins or ends) is approximately a stationary discrete time Markov chain with transition probabilities

(1) $$P\left(i,j\right)=\{\begin{array}{ll} 1, & \text{if}\;i=0,\;j=1,\\ p\left(i\right), & \text{if}\;i\geq 1,\;j=i+1,\\ 1-p\left(i\right), & \text{if}\;i\geq 1,\;j=i-1,\\ 0, & \text{otherwise,} \end{array}$$

where

$$p\left(k\right)=k!\left(\sum_{j=0}^{k}\frac{{\left(-1\right)}^{k-j}}{j!\theta^{k-j}}+\frac{{\left(-1\right)}^{k-1}e^{-\theta }}{\theta^{k}}\right),$$

for *k *≥ 1. The quantity *p(k) *is just the conditional probability that, for any fixed location *t*, a new read starts somewhere after *t *before any of the extended reads covering *t *end, given that there are *k *such extended reads.

Secondly, the random tree *T *constructed from an excursion of the coverage function above the level *h *is a Galton-Watson tree with generation-dependent geometric offspring distributions: the root is at height *h*, the probability a vertex at height *k *>*h *has *n *offspring (that is, it is connected to *n *vertices at height *k *+ 1) is *p(k)^n^*(1 - *p(k)*), *n *≥ 0, and these family sizes are independent. We could use this observation to simulate independent copies of *T *and to obtain a Monte-Carlo approximation of the distribution of the null distribution of ℳ(*T*). Instead, we simulate independent copies of the appropriate random lattice path and construct copies of *T *from them; that is, to construct a copy of the random lattice path we start at height *h*, we move to height *h *+ 1 at the first step, at succeeding steps we move up or down with respective probabilities *p(k) *and 1 - *p(k) *when we are at height *k*, and we stop when we return to height *h*.

Lastly, the expected number of vertices in such a tree is the expected length of an excursion above height *h *of a Markov chain with the transition probabilities (1). Denoting this quantity by *S*(*h*), we have $S\left(h\right)=\frac{1}{\pi \left(h\right)}$, where π is the stationary distribution of the Markov chain with state space {*h*, *h*+1, ... } that is obtained by taking the chain with the transition probabilities (1) and reflecting it at height *h*: intuitively, if an excursion above *h *has expected length *S(h)*, then the long term proportion of steps the reflected chain will be in state *h *is $\frac{1}{S\left(h\right)}$. Thus,π is the unique solution of the standard system of equilibrium equations

π(i)=π(i−1)P(i−1,i)+π(i+1)P(i+1,i),

for *i *>*h *with

π(h)=π(h+1)P(h+1,h)

subject to the normalization $\sum_{i}\pi \left(i\right)=1$ [11, §6.4].

#### Subdividing the genome into regions

As we remarked above, it is inappropriate to use the same rate *ρ *, perhaps estimated by $\frac{\#\;\text{of}\;\text{reads mapped}}{\text{length}\;\text{of genome}}$, for the entire genome. Instead, we subdivide the genome into homogeneous regions based on the input control and perform our analysis on each region separately. Given the input, we calculate a local rate function

$$\varsigma \left(t\right)=\frac{\#\;\text{of}\;\text{input tags starting in }I_{t}}{1000},$$

where *I_t _*is the interval of length 1000 centered at *t*. We then discretize *ζ *into a step function as follows. For each chromosome, we begin with the interval *I *= [1, *K*], where *K *is a user specified integer, and find the average of *ζ *over *I*. We extend *I*, adding nucleotides *K *+1, *K *+2, ..., *t*_0 _until *ζ *(*t*_0 _+1) differs from the computed average *ζ *by more than a fixed user specified value *D*. The next interval begins as [*t*_0 _+ 1, *t*_0 _+ *K*], and it is extended until jumps away from its average by more than *D*. For the human genome, we use *K *= 10, 000, but one could use a smaller *K *for shorter genomes. Additionally, we use *D *= 5. Once all the intervals are determined for all chromosomes, we round each average *ζ *to the nearest integer and define (disconnected) regions *R_j _*based on the intervals whose average *ζ *rounds to *j*. We calculate the local rate

$$p_{j}=\frac{\#\;\text{of}\;\text{tags in data originating in }R_{j}}{\sum_{∐I=R_{j}}\text{length}\left(I\right)}$$

for the data along *R_j_*.

#### Initial filtering of possible peaks

For each region *R*, we fix a height *h_R _*and obtain a collection of trees/possible peaks from the segments in the set

$$S=\{t\;\in R|f\left(t\right)\geq h_{R}\}$$

(a segment is a subset of S consisting of contiguous nucleotides). Care must be used when selecting *h_R_*. If *h_R _*is too low, then we will pick up trees that are so broad that it is impractical to approximate the null distribution of our test statistic using Monte-Carlo methods. Additionally, our called peaks will be very wide. On the other hand, if *h_R _*is too high, then we may not catch genuine peaks. We choose

$$h_{R}:=\max\;\left(\left\lceil \theta \right\rceil ,\min\;\left\{h|S\left(h\right)\leq C\right\}\right),$$

where *θ *is the estimated expected height of the coverage function on *R *and *C *is a user-specified parameter. Note that *h_R _*increases as *C *decreases. We use *C *= 7 in our analysis.

#### Identifying peaks and correcting for multiple hypotheses

For a homogeneous region *R*, consider a random variable obtained by evaluating our statistic ℳ on a tree built from an excursion of the coverage function above the level *h_R _*under the null model. Let *G_R_*(*m*) be the probability that such a random variable exceeds *m*. In order to approximate *G_R_*, we simulate 30,000 random trees with root at height *h_R _*via the method described above of simulating the associated lattice path.

We find the segments in the observed coverage function that correspond to excursions above *h_R _*that are at least 10 base pairs long. We build the lattice path and tree associated with each such excursion. We then compute the value ℳ(*T*) of our statistic ℳ for each such tree *T *and assign the 'p-value' *G_R_*(ℳ(*T*)) to *T*.

**Figure 3:**
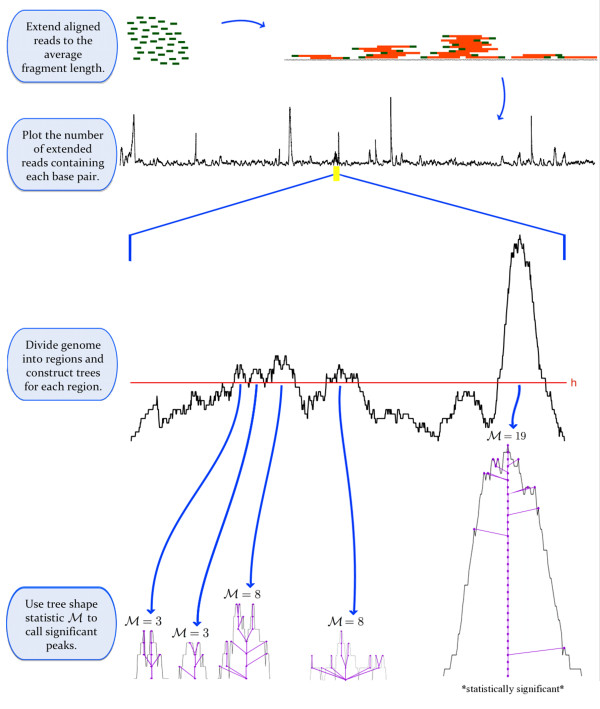
**An overview of our method**. In our method, aligned reads are extended to the average fragment length (for single end sequencing), and a coverage function records the number of extended reads containing each base pair. Trees capturing the shape of the coverage function are constructed and a tree shape statistic measuring the size of a maximal matching ℳ is computed. By comparison to a null model derived from the expected shape of random trees, significant peaks are identified.

With *α *= 0.01 as the significance level, we use a Benjamini-Hochberg correction [12,13] for multiple hypothesis testing as follows. We first take the 'p-values' for the *N *trees found on the entire genome, and order these probabilities from least to greatest *p*_(1) _≤ *p*_(2) _≤ ... ≤ *p*_(*N*)_. Let *J *be the largest *j *such that $p_{\left(j\right)}\leq \frac{j\alpha }{N}$. A tree *T *in a region *R *is a called as a peak provided $G_{R}\left(ℳ\left(T\right)\right)\leq \frac{J\alpha }{N}$. We merge two called peaks in bordering regions provided the gap between them is less than *L*. Figure 3 gives a pictorial sketch of our method.

### Testing

We tested T-PIC by predicting binding sites for publicly available data sets. Rather than comparing T-PIC to every possible peak caller, we identified PeakSeq [14] and MACS [15] based on previous studies [4,5] as being the best current programs, and restricted our comparisons to them.

#### Binding site prediction using published data sets

With our algorithm, we predicted binding sites for four transcription factors (with a total of 6 antibodies) for Drosophila melanogaster. We used published data from the Eisen lab [16] (available at the NCBI GEO database [17], accession GSE20369). Additionally, we predicted binding sites for the human genome for STAT1 using data from the Gerstein lab (available at [18]) and for FoxA1 using data from the Liu lab (available at [19]). Table 1 gives information on each sample used in our analysis. We compared our method to PeakSeq [14] and MACS [15] on each data set, and peaks were called with MACS and PeakSeq using the default parameters. Table 2 gives a summary of the peaks called by T-PIC, MACS, and PeakSeq.

**Table 1:** Samples used in comparison analysis

**Samples used in comparison analysis**.				
**Protein**	**Sample**	**# of Mapped Reads**	**# of Input Mapped Reads**	**Reference**

cad	D. melanogaster	4,695,843	5,275,977	[16]

gt	D. melanogaster	4,702,233	13,952,235	[16]

hb1	D. melanogaster	3,470,895	13,952,235	[16]

hb1	D. melanogaster	3,018,544	13,952,235	[16]

kr1	D. melanogaster	5,175,465	5,275,977	[16]

kr2	D. melanogaster	5,075,323	5,275,977	[16]

FoxA1	MCF7 cells	3,909,805	5,233,683	[15]

STAT1	Stimulated Hela S3 cells	26,731,492	19,476,469	[14]

The samples used in the Testing section are listed along with their references. Additionally, the numbers of mapped reads for the sample and for the input are given.

**Table 2:** Summary of called peaks.

**Summary of called peaks**.						
**Protein**	**Peak Caller**	**Mean Length**	**# of Peaks**	**% Found by T-PIC**	**% Found by MACS**	**% Found by PeakSeq**

cad	T-PIC	990.9	8136	100	64.0	91.4
	MACS	1659.6	4601	95.7	100	99.9
	PeakSeq	5278.3	11612	38.9	29.1	100

gt	T-PIC	896.1	4502	100	59.3	71.4
	MACS	1241.4	2929	85.6	100	89.3
	PeakSeq	16030.8	3497	48.4	38.8	100

hb1	T-PIC	978.5	7523	100	76.7	89.9
	MACS	1403.4	5640	93.9	100	99.9
	PeakSeq	876.3	12072	57.8	53.7	100

hb2	T-PIC	930.9	6392	100	75.6	87.4
	MACS	1321.2	4849	92.4	100	99.9
	PeakSeq	545	11037	54.5	52.3	100

						
kr1	T-PIC	883.0	11505	100	68.0	93.9
	MACS	1624.3	6490	98.3	100	99.9
	PeakSeq	5189.1	12924	45.9	33.8	100

kr2	T-PIC	884.0	11409	100	67.4	94.2
	MACS	1588.4	6393	98.3	100	100
	PeakSeq	5040.9	13540	43.9	31.5	100

FoxA1	T-PIC	510.7	17619	100	64.4	57.4
	MACS	394.1	13639	83.7	100	69.6
	PeakSeq	391.6	10320	97.8	91.1	100

STAT1	T-PIC	857.3	84465	100	36.8	62.5
	MACS	1342.3	29121	96.9	100	97.2
	PeakSeq	573.8	62124	86.8	51.5	100

A summary of predictions for bicoid (bcd), caudal (cad), giant (gt), hunchback antibody 1 (hb1), hunchback antibody 2 (hb2), knirps (kni), kruppel antibody 1 (kr1), and kruppel antibody 2 (kr2) is given.

**Figure 4:**
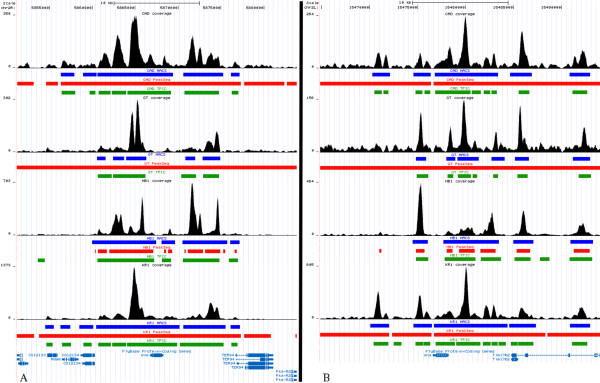
**Examples of predicted peaks for Drosophila melanogaster**. Peaks called by T-PIC, MACS, and PeakSeq for four transcription factors--caudal (cad), giant (gt), hunch-back antibody 1 (hb1), and kruppel antibody 1 (kr1)--in the even skipped (A) and snail (B) loci are shown. The coverage function for each protein is also plotted in the UCSC Genome Browser [20]. Peaks called by T-PIC are depicted with green bars, PeakSeq's peaks are in red, and the peaks called by MACS are shown in blue.

Figure 4 gives examples as to how the peaks called by T-PIC, MACS, and PeakSeq differed from each other. Called peaks for each of the D. melanogaster transcription factors in the even skipped (eve) and snail (sna) loci are shown in the UCSC genome browser [20]. The binding for these two well-characterized loci has been previously studied [21]. In many cases, our peaks subdivided those called by MACS and for 3 of the proteins, our peaks subdivided those called by PeakSeq in agreement with where binding occurs. We additionally demonstrate the prediction of a binding site for hunchback in the snail loci that both MACS and PeakSeq miss.

#### Validation of called peaks

To show that examples such as those above were significant and reproducible, we tested the peaks called by T-PIC, MACS, and PeakSeq for binding motif enrichment for each protein analyzed. We assigned an enrichment score to each set of called peaks using fold enrichment as follows: for each protein and peak caller, we created random intervals from the genome by selecting the same number of intervals with the same lengths from each chromosome as in the called peaks but with random starting locations. We then counted the number of occurrences of the binding motif in the called peaks and in the random intervals. The enrichment score is the ratio of the number of occurrences in the called peaks divided by the number of occurrences in the random intervals. By using random sequences of the same lengths, we accounted for increased binding motif counts that would occur by chance in longer sequences. We could therefore compare the enrichment scores between different peak callers. Table 3 shows the motif enrichment scores for each protein and peak caller. Overall, T-PIC performed favorably as measured by motif enrichment. The average enrichment score for T-PIC is 1.823, while MACS averages 1.520 and PeakSeq averages 1.468. Moreover, for 5 of the 8 samples, T-PIC outperformed both MACS and PeakSeq as measured by motif enrichment.

**Table 3:** Motif Enrichment

**Motif Enrichment**.				
**Protein**	**Binding Motif**	**T-PIC**	**MACS**	**PeakSeq**

cad	${\text{TTTAT}}_{\text{GA}}^{\text{TG}}$	0.805	0.971	0.895

gt	TTACGTAA	2.347	1.59	1.042

hb1	TTTTTT	1.673	1.61	1.572

hb2	TTTTTT	1.722	1.641	1.956

kr1	${}_{\text{A}}^{\text{G}}\text{ANGGGT}$	1.748	1.523	1.099

kr2	${}_{\text{A}}^{\text{G}}\text{ANGGGT}$	1.732	1.508	1.01

FoxA1	TGCATG	2.547	1.682	1.976

STAT1	TTCNNNGAA	1.454	1.633	2.196

The motif enrichment score is fold enrichment over random sequences. This score is listed for each protein and peak caller. The random sequences used have the same number and lengths as a given set of peaks, but the start site is randomly chosen from the chromosome. References containing the binding motifs are [25] (for cad, gt, hb, and kr), [26] (for FoxA1), and [27] (for STAT-1).

We then compared the called peaks to the results of independent qPCR experiments for STAT1 and FoxA1 proteins. For FoxA1, we used 26 true positives and 12 true negatives found in [22]. For STAT1, we used 20 true positive regions and 42 true negative regions found in [23]. T-PIC found 15 of 26 positives for FoxA1 and 18 of 20 positive regions for STAT1. MACS finds 14 of 26 positives for FoxA1 and 18 of 20 positive regions for STAT1. PeakSeq finds 13 of 26 positives for FoxA1 and 15 of 20 positive regions for STAT1. In terms of true negatives, T-PIC found 2 of 12 negatives for FoxA1 and 4 of 42 negative regions for STAT1, PeakSeq found 0 of 12 negatives for FoxA1 and 2 of 42 negative regions for STAT1, and MACS found 0 or 12 negatives for FoxA1 and 1 of 42 negative regions for STAT1. These results indicate that T-PIC has high sensitivity, finding more true positives than PeakSeq for both STAT1 and FoxA1 while finding more true positives than MACS for FoxA1. While our Specificity results on this experiment underperformed PeakSeq and MACS by analysis of prediction on true negatives, our results on the Drosophila experiment summarized in Table 1 show that we frequently call fewer peaks than PeakSeq. Moreover, both of the FoxA1 true negatives and 3 of the 4 STAT1 true negatives found by T-PIC pass PeakSeq's first pass of scoring. This means that they are potential peaks based on their height being extreme (and can therefore be considered "borderline" peaks). In general, accurate estimation of Specificity in peak calling is difficult because it is hard to rule out the validity of individual predicted peaks.

#### Robustness

To test for robustness against replicates, we used the two data sets for hunchback (antibodies 1 and 2) and kruppel (antibodies 1 and 2). For each antibody, we calculated the percentage of peaks that overlapped at least one peak from the other antibody for the same protein. The average percentage for T-PIC was 80.33, while MACS averaged 86.34 and PeakSeq averaged 78.37. We additionally analyzed the ChIP-Seq data for two sample lanes of the STAT1 data [18]. These two lanes came from replicate 2 and had a total of 8,938,780 mapped reads. We compared the predictions to those obtained using the full data set (a total of two replicates, six lanes, and 26,731,492 mapped reads). All three programs found fewer peaks with the smaller data set-- T-PIC predicted 72,778 peaks (13.8% fewer), MACS predicted 19,132 peaks (34.3% fewer), and PeakSeq found 32,232 peaks (48.1% fewer). Of the peaks found using replicate 2, 92.2% of T-PIC's called peaks overlapped peaks found using T-PIC and the entire data set. This compared favorably to both MACS (with 92.0%) and PeakSeq (with 95.1%). and suggests that T-PIC is as robust as other peak calling methods in terms of biological replicates.

Next, we tested for robustness against the input parameter *L *as during the size selection step, a researcher may not know the true average fragment length. Using the STAT1 data (having *L *= 200), we ran T-PIC with the additional *L *values: 150, 175, 225, and 250. On average, the peaks found using different *L *values overlapped 86.87% of the peaks called using *L *= 200. The lower values of *L *(150 and 175) resulted in more peaks than for *L *≥ 200 and we found a higher percentage of the *L *= 200 peaks than the higher values of *L *(225 and 250). In comparison, PeakSeq also used the input parameter *L*. On average 93.14% of the PeakSeq's peaks were found by the different *L *values. Although the true average fragment length for single end sequenced data may not be known, one could determine *L *if doing paired end sequencing. Our results suggest that this is a good idea regardless of which peak caller is used.

## Implementation

T-PIC is implemented in R [24] and calls a perl script that subdivides the genome into regions based on the input control. Our code is available at http://bio.math.berkeley.edu/tpic/, or upon request. Table 4 lists all parameters involved in our method, along with the parameter choices used in the Testing section.

**Table 4:** Parameters used in T-PIC

**Parameters used in T-PIC**.		
**Parameter**	**Brief Description**	**Value used in testing**

*L*	average fragment length	N/A(varies by experiment)

	minimum length of peak (in bp)	10

*α*	significance p-value	0.01

	width of interval used to calculate local rate *γ*(*t*)	1,000

*K*	minimum length of interval for discretizing *γ*	10,000 (human)

		5,000 (D. Melano.)

*D*	used in discretizing *γ*	5

*C*	using in selecting height *h*	7
	number of random trees per region in simulation	30,000

The parameters involved in the T-PIC algorithm are presented as well as the values used in the Testing section. Further details on each parameter may be found in the Algorithm section.

## Conclusions

We have developed a novel approach to the analysis of ChIP-Seq data, that aims to discover bound regions of DNA by topological analysis of read coverage functions. Our method-T-PIC-is fast and freely available, making it suitable for general use. The approach compares favorably to two popular peak callers: PeakSeq and MACS. We find the majority of their called peaks while detecting additional sites of binding. Although we have focused on ChIP-Seq in this paper, the approach we describe to call peaks could also be of use in the analysis of other sequence based assays like for instance CLIP-Seq for protein-RNA interactions.

## Authors' contributions

LP proposed the problem of using the shape of a putative peak to determine binding sites in ChIP-Seq. SNE developed the probability theory. VH explored ideas from topological data analysis, implemented the algorithm, and analyzed the ChIP-Seq data. VH, SNE and LP worked together to develop the peak calling algorithm, and all contributed to writing the manuscript. All authors read and approved the final manuscript.
